# CXCR7 ameliorates myocardial infarction as a β-arrestin-biased receptor

**DOI:** 10.1038/s41598-021-83022-5

**Published:** 2021-02-09

**Authors:** Masato Ishizuka, Mutsuo Harada, Seitaro Nomura, Toshiyuki Ko, Yuichi Ikeda, Jiaxi Guo, Satoshi Bujo, Haruka Yanagisawa-Murakami, Masahiro Satoh, Shintaro Yamada, Hidetoshi Kumagai, Yoshihiro Motozawa, Hironori Hara, Takayuki Fujiwara, Tatsuyuki Sato, Norifumi Takeda, Norihiko Takeda, Kinya Otsu, Hiroyuki Morita, Haruhiro Toko, Issei Komuro

**Affiliations:** 1 Department of Cardiovascular Medicine, Graduate School of Medicine, The University of Tokyo, 7-3-1 Hongo, Bunkyo-ku, Tokyo, 113-8655 Japan; 2 Department of Advanced Clinical Science and Therapeutics, Graduate School of Medicine, , The University of Tokyo, Tokyo, Japan; 3 Department of Advanced Translational Research and Medicine in Management of Pulmonary Hypertension, Graduate School of Medicine, The University of Tokyo, Tokyo, Japan; 4 The School of Cardiovascular Medicine and Sciences, King’s College London British Heart Foundation Centre of Excellence, London, UK

**Keywords:** Cell biology, Molecular biology, Cardiology

## Abstract

Most seven transmembrane receptors (7TMRs) are G protein-coupled receptors; however, some 7TMRs evoke intracellular signals through β-arrestin as a biased receptor. As several β-arrestin-biased agonists have been reported to be cardioprotective, we examined the role of the chemokine receptor CXCR7 as a β-arrestin-biased receptor in the heart. Among 510 7TMR genes examined, *Cxcr7* was the most abundantly expressed in the murine heart. Single-cell RNA-sequencing analysis revealed that *Cxcr7* was abundantly expressed in cardiomyocytes and fibroblasts. Cardiomyocyte-specific *Cxcr7* null mice showed more prominent cardiac dilatation and dysfunction than control mice 4 weeks after myocardial infarction. In contrast, there was no difference in cardiac phenotypes between fibroblast-specific *Cxcr7*-knockout mice and control mice even after myocardial infarction. TC14012, a specific agonist of CXCR7, significantly recruited β-arrestin to CXCR7 in CXCR7-expressing cells and activated extracellular signal-regulated kinase (ERK) in neonatal rat cardiomyocytes. *Cxcr7* expression was significantly increased and ERK was activated in the border zone of the heart in control, but not *Cxcr7* null mice. These results indicate that the abundantly expressed CXCR7 in cardiomyocytes may play a protective role in the heart as a β-arrestin-biased receptor and that CXCR7 may be a novel therapeutic target for myocardial infarction.

## Introduction

There are many seven-transmembrane receptors (7TMRs), such as adrenergic receptors and angiotensin II receptors, in the heart, and they play various critical roles in cardiac function and morphology^1,2^. Most 7TMRs are guanine nucleotide-binding protein-coupled receptors (GPCRs) and evoke intracellular signals through G proteins; however, it has been recently reported that atypical chemokine receptors (ACKRs) 1–5 are 7TMRs, but do not elicit typical G protein-mediated signaling^3^. The roles of ACKRs are largely unknown, and only ACKR3, also known as CXC chemokine receptor 7 (CXCR7), has been shown to evoke signals through β-arrestin^4^. Ligands that evoke signals through β-arrestin rather than G proteins are called β-arrestin-biased agonists and have recently attracted substantial attention as a new drug target. A β-blocker, carvedilol, has been reported to be very effective for the treatment of heart failure patients because it not only blocks adrenergic receptors, but also activates cardioprotective signals, including ERK, through β-arrestin^5^. A small compound, TRV120067, which binds to type 1 angiotensin II receptors and activates signals through β-arrestin rather than G proteins, ameliorated cardiac function in a murine model of dilated cardiomyopathy^6^.

Recently, the importance of chemokines and their receptors in cardiovascular diseases has been pointed out. For example, CC chemokine ligand 2 induced the accumulation of CC chemokine receptor 2-positive macrophages in a pressure-overload heart failure model^7^. CXC chemokine ligand 12 (CXCL12), also known as stromal cell-derived factor-1, is a natural ligand of CXCR4 and CXCR7^8^, and several studies have demonstrated that CXCL12 effects are mainly established through activation of CXCR4, but not CXCR7^9–11^. For example, CXCL12 protects the heart from hypoxia^9^ by recruiting bone marrow-derived cells^10^ and promoting neoangiogenesis^11^ via CXCR4. CXCR7 reportedly competes with CXCR4 for CXCL12 and inhibits the functions of CXCR4 as a decoy receptor^12^. However, a recent study has shown that CXCR7 itself can evoke intracellular signals through β-arrestin^4^, suggesting that it functions as an activator of β-arrestin-biased signaling induced by CXCL12, rather than as a decoy receptor. As CXCR7 has been reported to be expressed in the heart^13^, we examined its role as a β-arrestin-biased receptor in the heart.

## Results

### *Cxcr7* is the most abundantly expressed 7TMR in the murine heart, particularly, in cardiac myocytes and fibroblasts

**Figure 1:**
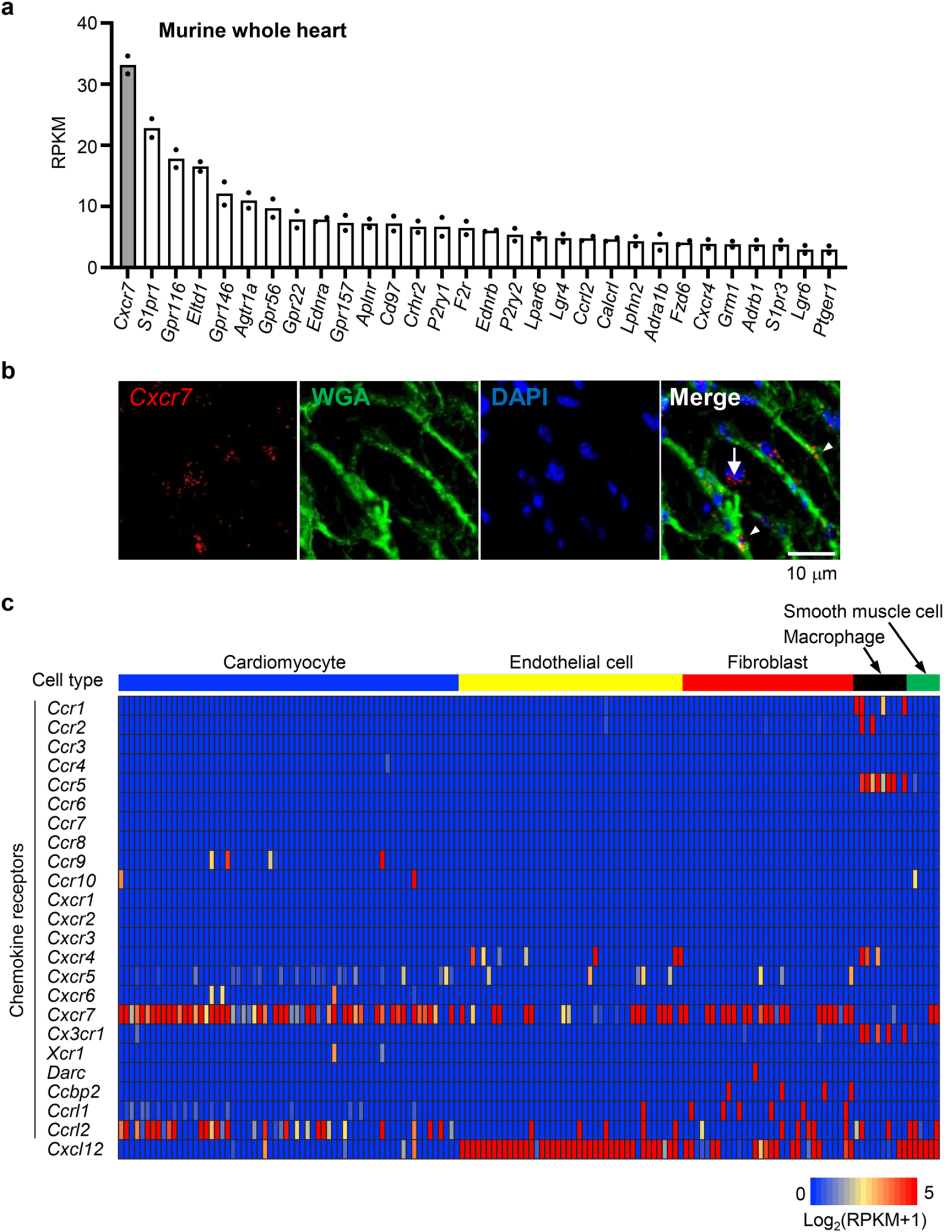
CXCR7 is the most abundantly expressed 7TMR in the murine heart. (**a**) Expression levels of 7TMRs in murine hearts determined by bulk RNA-seq. The 30 most strongly expressed 7TMRs are shown in the order of their mRNA expression levels. Data represent the mean expression levels from two mice. (**b**) smFISH for *Cxcr7* mRNA (red dots). Each dot indicates a *Cxcr7* mRNA molecule in a 10-μm-thick frozen heart section. Cell membranes were stained with Wheat Germ Agglutinin and Alexa Fluor 488 Conjugate to delineate cardiomyocytes and other cell types. White arrow, *Cxcr7* mRNA molecules in cardiomyocytes; arrowheads, *Cxcr7* mRNA molecules in the interstitial area (non-cardiomyocytes). (**c**) Single-cell RNA-seq profiles of chemokine receptors and CXCL12 expressed on cardiomyocytes (n = 64), endothelial cells (n = 42), fibroblasts (n = 32), macrophages (n = 10), and smooth muscle cells (n = 6) in the wild-type murine heart.

We first examined the expression levels of various 7TMRs in the murine heart by bulk RNA-sequencing (RNA-seq). Among 510 7TMR genes, *Cxcr7* was the most abundantly expressed in the murine heart (Fig. 1a). *Cxcr7* mRNA levels were three-fold higher than those of the type 1A angiotensin II receptor (*Agtr1a*) and tenfold higher than those of the β1-adrenergic receptor (*Adrb1*). Single-molecule fluorescence in situ hybridization (smFISH) of the murine heart revealed that *Cxcr7* was expressed in both cardiomyocytes and non-cardiomyocytes (Fig. 1b). We therefore performed single-cell RNA-seq analysis to examine mRNA levels of all 23 chemokine receptors in various heart cell types. Individual cells were identified as cardiomyocytes, endothelial cells, fibroblasts, macrophages, or smooth muscle cells by clustering analysis using cell type-specific gene modules constructed by weighted co-expression network analysis. The clustering results were confirmed based on analysis of cell-type specific gene markers, such as *Myh6*, *Myh7*, and *Mylk3* for cardiomyocytes, *Fabp4*, *Cav1*, and *Pecam1* for endothelial cells, *Dcn*, *Lum*, *Col1a1*, and *Col1a2* for fibroblasts, *C1qa*, *C1qb*, and *Csf1r* for macrophages, and *Rgs4*, *Kcnj8*, and *Tpm2* for smooth muscle cells (Supplementary Fig. S1). *Cxcr7* was abundantly expressed in nearly all cardiomyocytes (Fig. 1c). Expression levels of chemokine receptors other than *Cxcr7* and *Ccrl2* were very low or undetectable in cardiomyocytes. *Cxcr7* was also expressed in many cardiac fibroblasts, but it was not or hardly expressed in endothelial cells, smooth muscle cells, and macrophages (Fig. 1c). In contrast, *Cxcl12*, which encodes a ligand of CXCR7, was abundantly expressed in endothelial cells and smooth muscle cells, but very lowly in cardiomyocytes (Fig. 1c). *Cxcr4*, encoding another CXCL12 receptor, was not expressed in cardiomyocytes, but was expressed in endothelial cells and cardiac macrophages (Fig. 1c). These results indicated that CXCR7 is a major CXCL12 receptor in cardiomyocytes and that CXCR7 expressed in cardiomyocytes may play a more important role than previously anticipated.

### Loss of *Cxcr7* in cardiomyocytes exacerbates cardiac dilatation and systolic dysfunction after myocardial infarction

**Figure 2:**
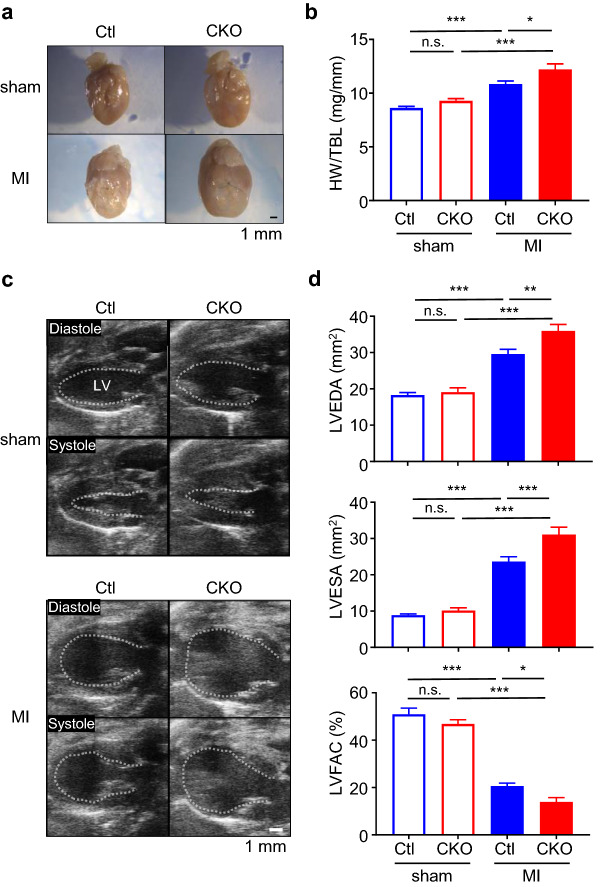
Deletion of *Cxcr7* in cardiomyocytes exacerbates post-infarction remodeling. (**a**) Representative images of infarcted hearts from Ctl and CKO mice 4 weeks after ligation of a left anterior descending artery to induce myocardial infarction. (**b**) Heart weight-to-tibia length ratio (HW/TBL) of infarcted mice. sham-Ctl, n = 10; sham-CKO, n = 9; MI-Ctl, n = 15; MI-CKO, n = 9. Data are shown as the mean ± SEM. Significance was calculated by one-way ANOVA followed by the Bonferroni procedure. *P < 0.05, ***P < 0.001. (**c**) Representative B-mode images of transthoracic echocardiography at diastole and systole of sham and infarcted hearts in Ctl and CKO mice. The dashed line indicates the endocardial surface of the left ventricular (LV) cavity. (**d)** Left ventricular end-diastolic area (LVEDA), end-systolic area (LVESA), and fractional area change (LVFAC) assessed by echocardiography 4 weeks after myocardial infarction. (sham-Ctl, n = 10; sham-CKO, n = 9; MI-Ctl, n = 15; MI-CKO, n = 9). Data are the mean ± SEM. Significance was calculated by one-way ANOVA followed by the Bonferroni procedure. *P < 0.05, **P < 0.01, ***P < 0.001.

As CXCR7 was found to be abundantly expressed in the heart, particularly in cardiomyocytes and fibroblasts, we examined its role in the heart using knockout mice (Supplementary Fig. S2a). As *Cxcr7* knockout mice are embryonic lethal^14–16^, we deleted *Cxcr7* specifically in cardiomyocytes or fibroblasts using the Cre/loxP system. Deletion of *Cxcr7* in cardiomyocytes resulted in a 77.9% ± 9.4% reduction in *Cxcr7* expression in the whole heart (Supplementary Fig. S2b), corroborating that CXCR7 is expressed mainly in cardiomyocytes among the various heart cell types. Since cardiomyocyte-specific *Cxcr7* null mice (αMHC-Cre^+/−^ CXCR7^flox/flox^; CKO mice) grew normally and showed no abnormal heart phenotype, we induced myocardial infarction by ligating a left anterior descending artery. CKO mice showed more prominent left ventricular enlargement and systolic dysfunction than control (Ctl) mice 4 weeks after myocardial infarction (Fig. 2a–d). In fibroblast-specific knockout mice (Col1a2-CreERT2^+/−^ CXCR7^flox/flox^; FKO mice), there was no significant reduction in *Cxcr7* expression in the whole heart (Supplementary Fig. S3a,b) and no difference in heart weight, left ventricular volume, and systolic function as compared with Ctl mice, even after myocardial infarction (Supplementary Fig. S3c,d).

### ERK is activated through CXCR7-β-arrestin signaling in cardiomyocytes

**Figure 3:**
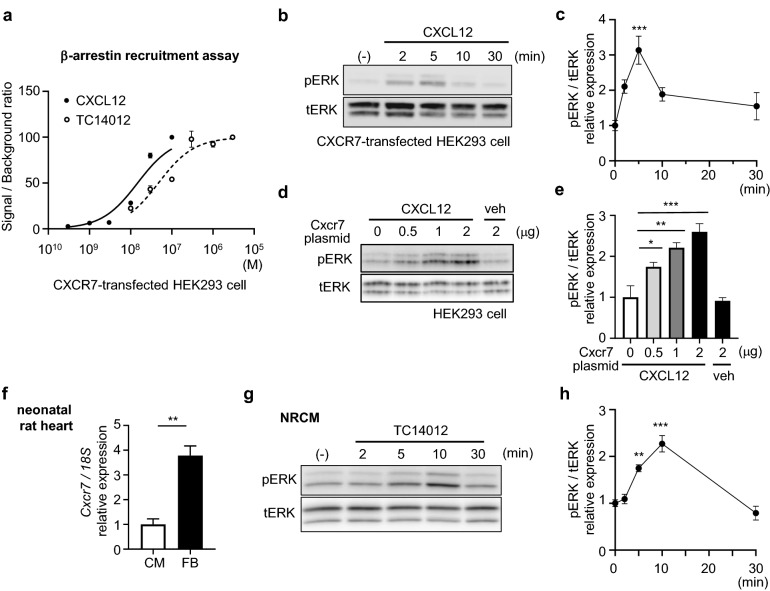
ERK is activated through CXCR7 in cardiomyocytes. (**a**) β-Arrestin recruitment assay of CXCR7 showing that CXCL12 and TC14012 induce coupling of CXCR7 with β-arrestin in a dose-dependent manner (EC_50_: 14.8 nM and 47.4 nM, respectively). Replicate samples are derived from independent HEK293 cells (n = 3). Data are shown as the mean ± SEM. (**b**) Immunoblot analysis of phosphorylated ERK (pERK) and total ERK (tERK) in HEK293 cells transfected with a CXCR7 expression plasmid at various time points after stimulation with CXCL12 (100 nM). (**c**) Quantitative data of the results shown in (**b**). n = 4. Data are shown as the mean ± SEM. Significance was calculated by ANOVA followed by the Bonferroni procedure; ***P < 0.001. Note that ERK was activated upon stimulation with CXCL12, and activity peaked at 5 min. (**d**) Immunoblot analysis of pERK/tERK in HEK293 cells transfected with various amounts of a CXCR7 expression plasmid with CXCL12 (100 nM) or vehicle (veh). Note that ERK was activated in a CXCR7 expression plasmid-dose-dependent manner under stable CXCL12 stimulation. (**e**) Quantitative data of the results shown in (**d**). n = 3. Data are shown as the mean ± SEM. Significance was calculated by one-way analysis of variance (ANOVA) followed by the Bonferroni procedure; *P < 0.05, **P < 0.01, ***P < 0.001. (**f**) *Cxcr7* mRNA expression in cardiomyocytes and fibroblasts from primary culture of neonatal rat hearts. Data are shown as the mean ± SEM. Significance was calculated by an unpaired *t*-test. **P < 0.01. (**g**) Immunoblot analysis of pERK and tERK in primary culture of NRCMs at various time points upon stimulation with the CXCR7-specific agonist, TC14012. (**h**) Quantitative data of the results shown in (**e**). n = 3. Data are shown as the mean ± SEM. Significance was calculated by ANOVA followed by the Bonferroni procedure; **P < 0.01, ***P < 0.001.

To unravel the mechanism underlying the protective effect of CXCR7 on the infarcted heart, we first performed *in-vitro* experiments to investigate whether CXCR7 acts as a β-arrestin-biased receptor. We overexpressed CXCR7 and β-arrestin in human embryonic kidney (HEK)293 cells and examined their coupling by measuring luminescence. CXCL12 dose-dependently induced β-arrestin recruitment to CXCR7, as anticipated (Fig. 3a). As β-arrestin activates ERK as a scaffold^17^, phosphorylation of ERK was examined. CXCL12 activated ERK in a CXCR7 expression plasmid-dose-dependent manner (Fig. 3b–e). We next used neonatal rat cardiomyocytes (NRCMs), which more abundantly express CXCR7 than cardiac fibroblasts do (Fig. 3f). Although CXCR4 expression was hardly detectable in cardiomyocytes (Fig. 1c), we used TC14012, a CXCR7-specific agonist^18^, instead of the natural ligand CXCL12 to eliminate the possibility of CXCL12-induced CXCR4 signal transduction in this experiment. We first examined the ability of TC14012 to act as a CXCR7 agonist; the results confirmed that TC14012 successfully recruited β-arrestin to CXCR7 in CXCR7-expressing cells (Fig. 3a). We then found that TC14012 significantly activated ERK in cultured cardiomyocytes, with a peak at 10 min after administration (Fig. 3g, h). Inhibition of β-arrestin signaling by barbadin^19^ abolished TC14012-induced ERK activation (Supplementary Fig. S4a,b), suggesting that CXCR7 might function as a β-arrestin-biased receptor in cardiomyocytes. We next investigated whether CXCR7 signaling can protect H9c2 cardiac cells from cell death. After confirming ERK activation by TC14012 in H9c2 cells (Supplementary Fig. S5a,b), cell death was induced by oxygen–glucose deprivation, which is widely used as an *in-vitro* model of myocardial ischemia. The mortality of H9c2 cells was 82.2% ± 4.8% after 24 h of anoxia in the presence of glucose 0.1 g/L (Supplementary Fig. S5c). Upon TC14012 administration, the mortality of H9c2 cells under the oxygen–glucose deprivation condition was modestly but significantly decreased (Supplementary Fig. S5d,e) when compared to vehicle treatment.

**Figure 4:**
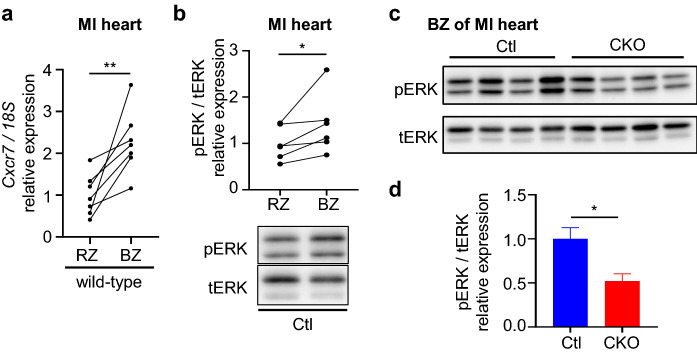
*Cxcr7* is upregulated concomitantly with ERK activation in the border zone of myocardial infarction. (**a**) *Cxcr7* mRNA levels in infarcted murine hearts. The heart tissue was dissected into two portions: a remote zone (RZ) and a border zone (BZ). Replicate samples were derived from heart tissues of wild-type mice (n = 7). Data are shown as paired data points. Significance was calculated by a paired *t*-test; **P < 0.01. (**b**) Immunoblot analysis of pERK and tERK in the RZ and BZ of infarcted heart. The bar chart shows the mean pERK-to-tERK ratio normalized to the value in the RZ. Samples were from infarcted heart tissues of Ctl mice (n = 6). Data are shown as paired data points. Significance was calculated by a paired *t*-test. *P < 0.05. (**c**) Immunoblot analysis of pERK and tERK in the BZ in infarcted hearts of Ctl and CKO mice. (**d**) Quantitative data of the results in Fig. 4c. Ctl, n = 6; CKO, n = 4. Data are shown as the mean ± SEM. Significance was calculated by an unpaired *t*-test. *P < 0.05.

We next examined *Cxcr7* expression and ERK activation in murine hearts after myocardial infarction. CXCR7 expression was significantly increased in the border zone than in the remote zone of infarcted hearts (Fig. 4a). ERK was concomitantly activated in the border zone as compared to the remote zone in Ctl mice (Fig. 4b), whereas such ERK activation was significantly attenuated in the hearts of CKO mice (Fig. 4c, d).

**Figure 5:**
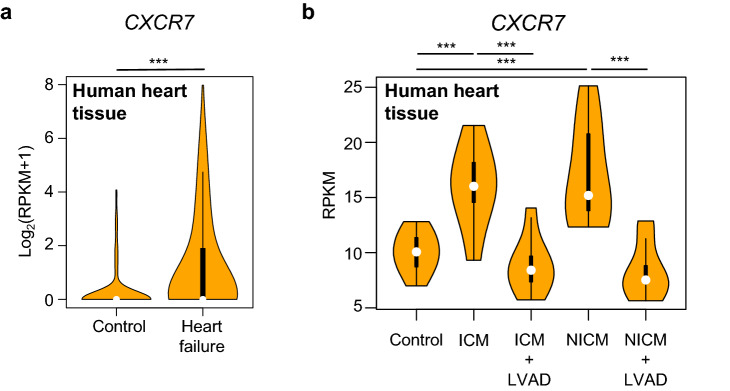
CXCR7 expression in cardiomyocytes is increased in heart failure patients. (**a**) Violin plots of *CXCR7* mRNA expression based on single-cardiomyocyte RNA-seq of human heart specimens obtained from control subjects (n = 156) and patients with heart failure (n = 678). Significance was calculated by the Mann–Whitney test; ***P < 0.001 (**b**) Violin plots of *CXCR7* mRNA expression based on bulk RNA-seq of human failing hearts before and after LVAD implantation. Analysis was performed using publicly available data^20^. Significance was calculated by ANOVA followed by the Bonferroni procedure; ***P < 0.001. ICM, ischemic cardiomyopathy; NICM, non-ischemic cardiomyopathy.

Finally, we examined the importance of CXCR7 in cardiomyocytes to determine its role in the pathophysiology of human heart failure. Single-cardiomyocyte RNA-seq of human heart specimens revealed higher *CXCR7* expression levels in cardiomyocytes of heart failure patients than in controls, suggesting that CXCR7 might be involved in the pathophysiology of heart failure in a clinical setting (Fig. 5a). To confirm these findings in human heart failure, we analyzed publicly available RNA-seq data of human heart failure^20^. In this study, RNA-seq raw data of paired nonischemic (n = 8) and ischemic (n = 8) human failing left ventricular samples collected before and after left ventricular assist device (LVAD) implantation and of nonfailing human left ventricle (n = 8) were reported. We confirmed that *CXCR7* expression was upregulated in failing hearts, regardless of the cause of heart failure (ischemic or nonischemic) (Fig. 5b). Interestingly, *CXCR7* upregulation was abolished upon LVAD implantation in both ischemic and non-ischemic cardiomyopathies, corroborating the association of heart failure with CXCR7.

## Discussion

CXCR7 has long been recognized as a decoy receptor of CXCR4, but has recently been appreciated as an important receptor in mediating normal biological and pathophysiological functions, including cardiac development. In postnatal mice, *Cxcr7* is expressed in cardiomyocytes and endothelial cells, and global *Cxcr7* deletion is lethal due to malformation of the cardiac valves^15,16^. However, the function of CXCR7 in the pathophysiology of adult heart remained elusive and its expression landscape, cell origin, or function, particularly in cardiomyocytes, had not been thoroughly investigated.

In this study, we focused on CXCR7 because of its abundant expression in the heart and its unique character as a 7TMR. *Cxcr7* was found to be the most abundant receptor gene expressed in the murine heart, among not only chemokine receptor genes, but also 7TMR genes. As a 7TMR, the β1-adrenergic receptor plays critical roles in the heart^21,22^; however, its expression level was tenfold lower than that of *Cxcr7*. Numerous chemokine receptors were found to be expressed in the heart, but *Cxcr7* showed substantially higher expression than other chemokine receptors in both cardiac myocytes and fibroblasts. This finding was corroborated by published RNA-seq data showing that *Cxcr7* expression was the highest in cardiomyocytes, followed by fibroblasts^23^. Interestingly, *Ccrl2* was also expressed in cardiomyocytes (Fig. 1c). CC chemokine receptor-like 2 (CCRL2) is another ACKR; it does not bind to chemokines, but to chemerin^24^. Its role in the heart has not been reported, but the fact that cardiomyocytes express ACKRs such as *Cxcr7* and *Ccrl2* rather than the majority of chemokine receptors warrants further investigation.

CXCL12 reportedly acts as a ligand for both CXCR4^3^ and CXCR7^8^. Several studies have indicated that CXCR4 plays central roles^9–11^, whereas CXCR7 solely inhibits CXCR4 activation by competing for CXCL12 as a decoy receptor^12^. In the heart, however, *Cxcr4* was not expressed in cardiomyocytes, and it was expressed at low levels in endothelial cells and cardiac macrophages. Furthermore, it has been reported that CXCR7 has a tenfold higher affinity for CXCL12 than CXCR4^[13]^ possibly because both receptors have different binding sites for CXCL12^25^. These results strongly suggest that CXCR7, not CXCR4, plays major roles in the heart as a CXCL12 receptor.

ACKRs have no typical G protein-binding DRYLAIV motif and do not evoke typical intracellular signaling through G proteins^26^. Therefore, the signals and roles of many ACKRs are expected to differ from those of GPCRs, but they are largely unknown, and many ACKRs are thought to be decoy receptors^3^. However, among the ACKRs, only ACKR3 (i.e., CXCR7) has been reported to activate intracellular signaling by recruiting β-arrestin as a biased receptor^4^. Although CXCR7 is regarded as a β-arrestin-biased receptor that is unable to evoke G-protein signaling because it has a distinct DRYLSIT motif, some studies have reported G-protein signaling via CXCR7. Odemis et al*.* provided evidence that CXCR7 in astrocytes signals through Gαi/o coupling, and Nguyen et al. showed ligand binding to CXCR7 in HEK293 cells that did not result in the activation of typical GPCR signaling pathways via Gα subunit, but in the activation of GRK2 via βγ subunits. As the involvement of G protein in CXCR7 signaling is still controversial and our main focus was β-arrestin, the β-arrestin inhibitor barbadin was used to evaluate whether or not CXCR7-induced ERK activation depended on β-arrestin. Indeed, the activation of ERK by TC14012 was completely suppressed upon barbadin treatment, suggesting that CXCR7 signaling in cardiomyocytes is β-arrestin-dependent (Supplementary Fig. S4a,b).

There are numerous reports on biased agonists. Biased agonism occurs when two ligands share one receptor, but transmit different signals^27^. For example, angiotensin-(1-7), a metabolite of angiotensin II, binds to type 1 angiotensin II receptor, but fails to activate G proteins, and selectively promotes β-arrestin activation compared with angiotensin II^28^. Angiotensin-(1-7) attenuates cardiac hypertrophy and is expected to have cardioprotective effects^29^. However, there are a few natural β-arrestin-biased agonists and very few 7TMRs that activate intracellular signaling only through β-arrestin rather than through G proteins. In this study, we clearly showed that CXCL12 recruits β-arrestin to CXCR7 and activates ERK both in vitro and in vivo. TC14012, a specific CXCR7 agonist, also recruited β-arrestin, albeit to a slightly lower extent than CXCL12 (Fig. 3a), but to a greater extent than AMD3100 reportedly did^18,30^. TC14012 rather than the natural ligand CXCL12 was used in *in-vitro* experiments throughout this study as a CXCR7 ligand to allow us to focus specifically on CXCR7 and β-arrestin signaling rather than CXCR4 signaling. TC14012 can also be used in *in-vivo* mouse experiments, and it will be interesting to conduct gain-of-function studies of CXCR7 using TC14012 or CXCR7 transgenic mouse in future.

CKO mice showed more prominent cardiac abnormalities, such as cardiac dilatation and dysfunction, after myocardial infarction than Ctl mice, indicating that CXCR7 in cardiomyocytes protects the heart from cardiac dysfunction after ischemia. One possible cardioprotective mechanism of CXCR7 in vivo may be that it has a beneficial effect on cell survival under ischemia. Our *in-vitro* study demonstrated that the mortality of H9c2 cells after oxygen–glucose deprivation was modestly but significantly decreased upon TC14012 administration, corroborating that CXCR7 improves cell survival (Supplementary Fig. S5d,e).

There have been many contradictory reports on the roles of ERK in cardiac hypertrophy and heart failure^31–35^. ERK has been reported to be essential for cardiomyocyte survival^31^ and to be involved in the development of physiological hypertrophy^32,33^ whereas other studies have indicated that ERK activation induces maladaptive hypertrophy, resulting in heart failure with fibrosis^34,36,37^. It has been recently reported that ERK-dependent maladaptive hypertrophic signaling is initiated by phosphorylation of the Thr188 residue, which in turn promotes nuclear localization of ERK and transcription of numerous genes involved in maladaptive hypertrophy^34,35,38^. In contrast, ERK activated by physiological hypertrophic signals such as β-arrestin^39^ has been reported to remain in the cytoplasm, facilitating the interaction of signaling components and stimulating cardioprotective signal transduction^40,41^. These results strongly suggest that ERK activated by CXCR7 through β-arrestin remains in the cytoplasm and protects the heart after myocardial infarction.

As CXCR7 expression is increased in human failing hearts, it is speculated that it may have a cardioprotective effect on the human heart. In the public RNA-seq data from paired failing cardiac tissue samples pre and post LVAD implantation, we found genes, including *NPPA*, *NPPB*, *SLC6A6*, and *BEX1*, which were upregulated before LVAD implantation and normalized in parallel with *CXCR7* after LVAD implantation*.* NPPA, NPPB, and SLC6A6 have been reported as cardioprotective molecules^42,43^, and BEX1 as a detrimental one^44^. Although CXCR7 seemed to promote cell survival under the ischemic condition in vitro*,* it is unclear whether the upstream of CXCR7 regulation is shared with other cardioprotective molecules, such as NPPA, NPPB, or SLC6A6. *CXCR7* expression reportedly is increased upon tissue hypoxia via VEGF^45^ or in the inflammatory response via NF-kB induction^46^ and therefore, it is plausible that both hypoxia and an inflammatory response occur within the border zone of the infarcted heart, thereby enhancing CXCR7 expression to attenuate cell death (Fig. 4a). Although CXCR7 is also expressed in cardiac fibroblasts, which are implicated in healing or fibrosis after myocardial infarction^47^, we observed no difference between FKO mice and Ctl mice even after myocardial infarction. While we do not rule out the possibility that CXCR7 in other cell types plays a role in the heart, our study clearly indicates that CXCR7 abundantly expressed in cardiomyocytes plays a critical role in the heart and that it might be a novel treatment target for myocardial infarction.

## Methods

### Mice and surgical procedures

All animal studies were approved by the University of Tokyo Ethics Committee for Animal Experiments (approval no.: # medicine P15-033) and adhered strictly to animal experimental guidelines and the ARRIVE guidelines. All mice were on the C57BL/6 background. Wild-type mice were purchased from CLEA Japan (Tokyo, Japan). We used the Cre/loxP system to generate conditional *Cxcr7*-knockout mice. CXCR7^flox/flox^ mice were provided by Prof. Fabienne Mackay (University of Melbourne, Australia). αMHC-Cre mice (stock no.: #009074, stock name: Tg [Myh6-cre]1Jmk/J) and Col1a2-CreERT2 mice (stock no.: 029567, stock name: Tg(Col1a2-cre/ERT,-ALPP)7Cpd) were purchased from The Jackson Laboratory (Bar Harbor, ME, USA). Myocardial infarction was surgically induced in 10–12-week-old male knockout mice (Cre^+/−^ CXCR7^flox/flox^) and their control littermates (Cre^−/−^ CXCR7^flox/flox^) by left anterior descending artery ligation, as previously described^48^. Col1a2-CreERT2 mice were operated two weeks after oral tamoxifen (T5648; Sigma, St. Louis, MO, USA)) administration (80 mg/kg, 4 days). Transthoracic echocardiography was performed using the Vevo2100 ultrasound system (FUJIFILM VisualSonics, Tokyo, Japan). B-mode echocardiography of the left ventricle was recorded in arousal in a parasternal long-axis view. Left-ventricular end-diastolic area, end-systolic area, and fractional area change were measured according to a previous report^49^. M-mode echocardiography of the basal left ventricle was recorded and left-ventricular end-diastolic dimension, end-systolic dimension, and fractional shortening were measured. The heart tissue was dissected into two portions, i.e., a remote zone at the base and a border zone at the middle of the border of the infarct area, under a stereomicroscope. Samples from these zones used for RNA and protein extraction were collected at 6 h and 24 h after ligation, respectively.

### NRCM and fibroblast isolation

NRCMs and neonatal rat fibroblasts were prepared from 1-day-old Wistar rats (Takasugi Experimental Animal Supply Co., Saitama, Japan), as previously described^50^. Briefly, cardiac cells were dispersed by digestion with collagenase type II (Worthington Biochemical, Lakewood, NJ, USA) and incubated at 37 °C for 80 min to divide the supernatant cells, which are rich in cardiomyocytes, and the attached cells, which are rich in cardiac fibroblasts. The supernatant NRCMs were cultured on gelatin-coated dishes at 37 °C in Dulbecco’s modified Eagle’s medium (DMEM; Nacalai Tesque, Kyoto, Japan) supplemented with 10% fetal calf serum for 36 h and confirmed on the basis of beating activity. After 12 h of starvation, the NRCMs were treated with TC14012 (Tocris Bioscience, Bristol, UK). Attached cells rich in cardiac fibroblasts were subcultured three times to increase their purity. We added vehicle (DMSO) or barbadin (MedChemExpress, New Jersey, USA) 30 min before stimulation.

### H9c2 cells and oxygen–glucose deprivation

H9c2 cells (ATCC CRL-1446, Manassas, VA, USA) were stimulated with TC14012 (30 μM) after 48 h of starvation to examine ERK activation. We used the BIONIX-1 hypoxic culture kit (Sugiyamagen, Tokyo, Japan) to establish an oxygen–glucose deprivation model in H9c2 cells as previously described^51^. Briefly, the medium was changed to DMEM without fetal bovine serum and glucose (A1443001, Gibco) supplemented with 0.1 g/L glucose and vehicle or TC14012 (3 μM). The cells were incubated in a closed chamber with an oxygen absorber and an oxygen monitor. The concentration of oxygen rapidly decreased to 0%. The chamber was maintained at 37 °C for 24 h. Then, the cells were stained using the ReadyProbes Cell Viability Imaging Kit (Thermo Fisher Scientific, Waltham, MA, USA) according to manufacturer’s instructions and imaged using an Operetta High-Content Imaging System (PerkinElmer). Mortality was quantified by counting SYTOX-positive dead nuclei in all Hoechst-positive nuclei.

### HEK293 cells and CXCR7 transfection

HEK293 cells (ATCC CRL-11268) were used for the β-arrestin recruitment assay, as previously described^52^. In brief, HEK293 cells were transfected with human β-arrestin-2-ω and human CXCR7-α. The cells were stimulated with human CXCL12 (R&D Systems, Minneapolis, MN, USA) or TC14012 at 37 °C for 90 min and then, Gal-Screen (Thermo Fisher Scientific) was added and the cells were further incubated at 25 °C for 90 min. Luminescence was measured in an ARVO X3 plate reader (PerkinElmer, Waltham, MA, USA). In addition, HEK293 cells were transfected with a rat CXCR7 expression plasmid using Lipofectamine 3000 (Thermo Fisher Scientific). The CXCR7 expression plasmid was synthesized by amplifying a rat *Cxcr7* mRNA sequence (RefSeq: NM_053352.1) with a FLAG tag (forward primer: 5′-TTTTGCGGCCGCGCCACCATGGATTACAAGGACGATGACGACAAGGGAGGAGGCTCCGATGTGCATCTGTTTGAC-3′, reverse primer: 5′-TTTTAAGCTTTCACTTGGTGTTCTGCTC-3′) from cDNA prepared from total RNA isolated from neonatal rat heart and inserting it in pShuttle-CMV (#16403, Addgene, Watertown, MA, USA). The cells were cultured for 36 h. After 12 h of starvation, the cells were stimulated with CXCL12.

### Sodium dodecyl sulfate polyacrylamide gel electrophoresis (SDS-PAGE) and immunoblotting

Western blotting was performed as previously described^53^. Tissues and cells were homogenized in ice-cold RIPA buffer containing protease inhibitor (cOmplete Protease Inhibitor Cocktail, Merck) and phosphatase inhibitor (PhosSTOP, Sigma). Supernatants were collected after centrifugation and mixed with an equal amount of loading buffer (125 mM Tris–HCl pH 6.8, 30% glycerol, 10% SDS, and 0.6 M DTT). After heat denaturation, the protein samples were subjected to SDS-PAGE using 10% acrylamide gels and transferred to polyvinylidene difluoride membranes (Millipore, Billerica, MA, USA). The membranes were incubated with primary antibodies against phospho-p44/42 MAPK (ERK1/2) (Thy202/Thy204) (9101S; Cell Signaling Technology, Danvers, MA, USA), p44/42 MAPK (ERK1/2) (9102S, Cell Signaling), and actin (MA5-11869; Thermo Fisher Scientific), and then with secondary antibodies against rabbit IgG (7074S; Cell Signaling Technology) and mouse IgG (NA931; GE Healthcare, Little Chalfont, UK). Signals were detected with the Pierce ECL Prime system (GE Healthcare) in a LAS4000 instrument (GE Healthcare). Band densities were analyzed using the ImageJ software (National Institutes of Health, Bethesda, MD, USA).

### RNA extraction and quantitative reverse transcription-quantitative (RT-q)PCR

Total RNA was extracted from tissues and cells using TRIzol reagent (Thermo Fisher Scientific) and was reverse-transcribed into cDNA using a reverse transcriptase (ReverTra Ace; TOYOBO, Osaka, Japan). qPCR was performed for 40 cycles using FastStart Essential DNA Green Master (Roche, Basel, Switzerland) and the following primers: *18S* (mouse, rat), forward primer: 5′-CTTAGAGGGACAAGTGGCG-3′, reverse primer: 5′-ACGCTGAGCCAGTCAGTGTA-3′; *Cxcr7* (mouse), forward primer: 5′-AGCCTGGCAACTACTCTGACA-3′, reverse primer: 5′-GAAGCACGTTCTTGTTAGGCA-3′; *Cxcr7* (rat), forward primer: 5′-ATCTTGAACCTGGCCATTGC-3′, reverse primer: 5′-TGTGTGATCTTGCACGTGAG-3′; *Cxcl12* (rat), forward primer: 5′-CAGAGCCAACGTCAAACATCTG-3′, reverse primer: 5′-TTCGGGTCAATGCACACTTG-3′; *Cxcr4* (rat), forward primer: 5′-CTCCAAGCTGTCACACTCCA-3′, reverse primer: 5′-TCCCCACGTAATACGGTAGC-3′. Data were analyzed using QuantStudio5 (Thermo Fisher Scientific). Relative gene expression was analyzed using the relative standard curve method^54^.

### Mouse bulk and cardiomyocyte or non-cardiomyocyte single-cell RNA-seq

Eight-week-old wild-type male mice were used for sequencing. For bulk RNA-seq, total RNA was extracted from mouse heart tissues (n = 2) using TRIzol reagent (Thermo Fisher Scientific). Purified RNA was reverse-transcribed by oligo-dT-primed first-strand synthesis using Superscript III (Thermo Fisher Scientific). PolyA + RNA was purified using the TruSeq Sample Purification Kit (Illumina, San Diego, CA, USA) and was sequenced on the Illumina GA II instrument.

For cardiomyocyte single-cell RNA-seq, cardiomyocytes were isolated from the left-ventricular free wall of the mouse heart (n = 2) using Langendorff perfusion and were used for single-cell cDNA library synthesis, as described previously^55^. Rod-shaped live cardiomyocytes (viability ≥ 80%) were collected immediately after isolation with a 0.2–2-µL pipette (sample volume, 0.5 µL) and incubated in lysis buffer. Single-cell cDNA libraries were generated using the Smart-seq2 protocol^56^, and the efficiency of reverse transcription was assessed by examining the threshold cycle (Ct) values of control genes (*Tnnt2*, *Cox6a2*) by RT-qPCR using a CFX96 Real-Time PCR Detection System (Bio-Rad, Hercules, CA, USA) and by examining the distribution of cDNA fragment lengths using LabChip GX (Perkin Elmer) and/or TapeStation 2200 (Agilent Technologies, Santa Clara, CA, USA). The libraries were sequenced using the HiSeq 2500 System (Illumina).

For non-cardiomyocyte single-cell RNA-seq, mouse hearts (n = 2) were minced and enzymatically dissociated using 2 mg/ml collagenase type II (Worthington Biochemical), 1 mg/ml dispase (Roche), and DNase I (Roche), with five cycles of digestion at 37 °C for a total of 40 min. After removing the cardiomyocytes using a 40-μm cell strainer (Greiner Bio-One, Kremsmünster, Austria), the cells were stained using the Zombie Aqua Fixable Viability Kit (BioLegend, San Diego, CA, USA), and live cells were collected by fluorescence-activated cell sorting in a FACS BD Jazz instrument (BD Biosciences, Franklin Lakes, NJ, USA). Single-cell cDNA library construction and sequencing were conducted as described for single-cardiomyocyte RNA-seq.

Reads were mapped to the mouse genome (mm9) using Bowtie (version 1.1.1)^57^ with the parameters “-S -m 1 -l 36 -n 2 mm9.” Reads per kilobase million (RPKM) values were calculated from reads mapped to the nuclear genome using DEGseq (version 1.8.0)^58^. Cells with RPKM > 0.1 for more than 5000 genes (for cardiomyocytes) or 2000 genes (for non-cardiomyocytes) were used for subsequent analysis. For bulk RNA-seq, the list of mouse 7TMRs to be analyzed was based on the protein database, UniProtKB/Swiss-Prot^59^. For non-cardiomyocytes, weighted co-expression network analysis using the WGCNA R package^60^ was applied to detect cell-type-specific gene modules, which were used for graph-based clustering using the buildSNNGraph function in the R package scran^61^. Annotated cell types were confirmed on the basis of cell-type-specific gene expression profiles (Supplementary Fig. S1).

### Human bulk and cardiomyocyte single-cell RNA-seq

To analyze the role of CXCR7 in cardiomyocytes in the pathophysiology of heart failure, we determined *CXCR7* expression levels from bulk RNA-seq data of human heart specimens published in the NCBI GEO database (GSE46224)^20^. This study analyzed paired nonischemic (n = 8) and ischemic (n = 8) human failing left ventricular samples collected before and after LVAD implantation and from nonfailing human left ventricles (n = 8).

Cardiomyocyte single-cell RNA-seq of human heart specimens was performed after obtaining approval from the ethics committee of the University of Tokyo (G-10032). All procedures were conducted according to the Declaration of Helsinki, and all patients provided written informed consent before taking part in the study. Heart tissue samples from control subjects (n = 2) and patients with heart failure (n = 22) were used. Heart tissues were obtained immediately after death due to non-cardiac causes (normal cardiac function) for control subjects and during LVAD surgery or heart transplantation for patients with heart failure. Immediately after heart tissue collection, rod-shaped live cardiomyocytes were isolated^62^ and incubated in a lysis buffer. Single-cell cDNA library construction was conducted as described above for mouse single-cell RNA-seq, and the efficiency of reverse transcription was assessed by examining the Ct values of a control gene (*TNNT2*) by qRT-PCR using a CFX96 Real-Time PCR Detection System and by examining the distribution of cDNA fragment lengths using the LabChip GX and/or TapeStation 2200. The libraries were sequenced using the HiSeq 2500 System. Reads were mapped to the human genome (hg19) using Bowtie^57^ with the parameters “-S -m 1 -l 36 -n 2 hg19.” RPKM values were calculated from reads mapped to the nuclear genome using DEGseq (version 1.8.0)^58^. Single-cell transcriptomes in which > 5000 genes were detected (RPKM > 0.1) were used for analysis (n = 156 for control subjects and n = 678 for patients with heart failure).

### smFISH

smFISH was performed using RNAScope (Advanced Cell Diagnostics, Newark, CA, USA) and a probe against *Cxcr7* mRNA (482561; Advanced Cell Diagnostics), as previously described^63^. Frozen heart sections (10 μm) were obtained from 8-week-old mice. They were co-stained with Wheat Germ Agglutinin, Alexa Fluor 488 Conjugate (Thermo Fisher Scientific) and ProLong Gold Antifade with DAPI (Life Technologies, Carlsbad, CA, USA). Images were obtained using the IN Cell Analyzer 6000 (GE Healthcare).

### Statistical analysis

Data are reported as the mean ± standard error of the mean (SEM). Statistical analysis was conducted using GraphPad Prism (GraphPad Software, Inc., La Jolla, CA, USA). An unpaired *t*-test was used for unpaired two-group comparisons and a paired *t*-test was used for paired two-group comparisons. For non-normally distributed samples, a non-parametric Mann–Whitney test was performed. Multiple-group comparisons were conducted by one-way analysis of variance (ANOVA) followed by the Bonferroni procedure. Significance was considered at *P* < 0.05, unless otherwise indicated.

## Supplementary Information

Supplementary Information.
