# Author Correction: CXCR7 ameliorates myocardial infarction as a β-arrestin-biased receptor

**DOI:** 10.1038/s41598-021-91788-x

**Published:** 2021-06-07

**Authors:** Masato Ishizuka, Mutsuo Harada, Seitaro Nomura, Toshiyuki Ko, Yuichi Ikeda, Jiaxi Guo, Satoshi Bujo, Haruka Yanagisawa‑Murakami, Masahiro Satoh, Shintaro Yamada, Hidetoshi Kumagai, Yoshihiro Motozawa, Hironori Hara, Takayuki Fujiwara, Tatsuyuki Sato, Norifumi Takeda, Norihiko Takeda, Kinya Otsu, Hiroyuki Morita, Haruhiro Toko, Issei Komuro

**Affiliations:** 1 Department of Cardiovascular Medicine, Graduate School of Medicine, The University of Tokyo, 7‑3‑1 Hongo, Bunkyo‑ku, Tokyo, 113‑8655 Japan; 2 Department of Advanced Clinical Science and Therapeutics, Graduate School of Medicine, The University of Tokyo, Tokyo, Japan; 3 Department of Advanced Translational Research and Medicine in Management of Pulmonary Hypertension, Graduate School of Medicine, The University of Tokyo, Tokyo, Japan; 4 The School of Cardiovascular Medicine and Sciences, King’s College London British Heart Foundation Centre of Excellence, London, UK

Correction to: *Scientific Reports* 10.1038/s41598-021-83022-5, published online 09 February 2021

This Article contains an error in Figure 3F, where the x-axis labels ‘CM’ and ‘FB’ were reversed.

Furthermore, the legend of Figure 3,

“ERK is activated through CXCR7 in cardiomyocytes. (**a**) β-Arrestin recruitment assay of CXCR7 showing that CXCL12 and TC14012 induce coupling of CXCR7 with β-arrestin in a dose-dependent manner (EC_50_: 14.8 nM and 47.4 nM, respectively). Replicate samples are derived from independent HEK293 cells (n = 3). Data are shown as the mean ± SEM. (**b**) Immunoblot analysis of phosphorylated ERK (pERK) and total ERK (tERK) in HEK293 cells transfected with a CXCR7 expression plasmid at various time points after stimulation with CXCL12 (100 nM). (**c**) Quantitative data of the results shown in (**b**). n = 4. Data are shown as the mean ± SEM. Significance was calculated by ANOVA followed by the Bonferroni procedure; ***P < 0.001. Note that ERK was activated upon stimulation with CXCL12, and activity peaked at 5 min. (**d**) Immunoblot analysis of pERK/tERK in HEK293 cells transfected with various amounts of a CXCR7 expression plasmid with CXCL12 (100 nM) or vehicle (veh). Note that ERK was activated in a CXCR7 expression plasmid-dose-dependent manner under stable CXCL12 stimulation. (**e**) Quantitative data of the results shown in (**d**). n = 3. Data are shown as the mean ± SEM. Significance was calculated by one-way analysis of variance (ANOVA) followed by the Bonferroni procedure; *P < 0.05, **P < 0.01, ***P < 0.001. (**f**) *Cxcr7* mRNA expression in cardiomyocytes and fibroblasts from primary culture of neonatal rat hearts. Data are shown as the mean ± SEM. Significance was calculated by an unpaired *t*-test. **P < 0.01. (**g**) Immunoblot analysis of pERK and tERK in primary culture of NRCMs at various time points upon stimulation with the CXCR7-specific agonist, TC14012. (**h**) Quantitative data of the results shown in (**e**). n = 3. Data are shown as the mean ± SEM. Significance was calculated by ANOVA followed by the Bonferroni procedure; **P < 0.01, ***P < 0.001.”

should read:

“ERK is activated through CXCR7 in cardiomyocytes. (**a**) β-Arrestin recruitment assay of CXCR7 showing that CXCL12 and TC14012 induce coupling of CXCR7 with β-arrestin in a dose-dependent manner (EC_50_: 14.8 nM and 47.4 nM, respectively). Replicate samples are derived from independent HEK293 cells (n = 3). Data are shown as the mean ± SEM. (**b**) Immunoblot analysis of phosphorylated ERK (pERK) and total ERK (tERK) in HEK293 cells transfected with a CXCR7 expression plasmid at various time points after stimulation with CXCL12 (100 nM). (**c**) Quantitative data of the results shown in (**b**). n = 4. Data are shown as the mean ± SEM. Significance was calculated by ANOVA followed by the Bonferroni procedure; ***P < 0.001. Note that ERK was activated upon stimulation with CXCL12, and activity peaked at 5 min. (**d**) Immunoblot analysis of pERK/tERK in HEK293 cells transfected with various amounts of a CXCR7 expression plasmid with CXCL12 (100 nM) or vehicle (veh). Note that ERK was activated in a CXCR7 expression plasmid-dose-dependent manner under stable CXCL12 stimulation. (**e**) Quantitative data of the results shown in (**d**). n = 3. Data are shown as the mean ± SEM. Significance was calculated by one-way analysis of variance (ANOVA) followed by the Bonferroni procedure; *P < 0.05, **P < 0.01, ***P < 0.001. (**f**) *Cxcr7* mRNA expression in cardiomyocytes and fibroblasts from primary culture of neonatal rat hearts. Data are shown as the mean ± SEM. Significance was calculated by an unpaired *t*-test. **P < 0.01. (**g**) Immunoblot analysis of pERK and tERK in primary culture of NRCMs at various time points upon stimulation with the CXCR7-specific agonist, TC14012. (**h**) Quantitative data of the results shown in (**g**). n = 3. Data are shown as the mean ± SEM. Significance was calculated by ANOVA followed by the Bonferroni procedure; **P < 0.01, ***P < 0.001.”

The correct Figure 3 and accompanying legend appear below as Figure 1.

**Figure 1:**
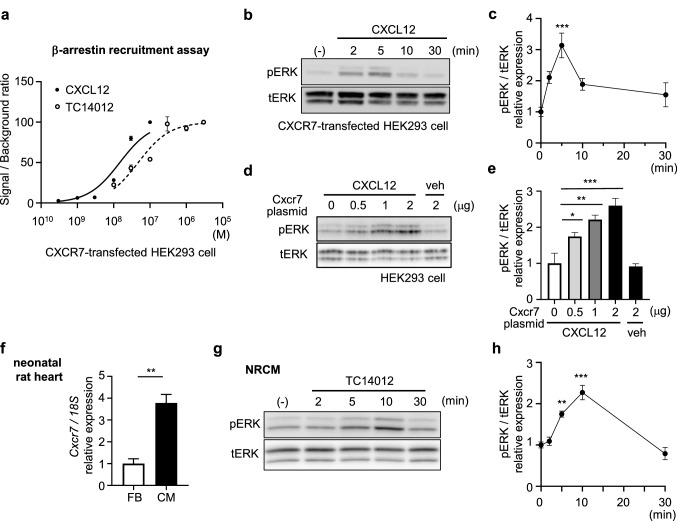
ERK is activated through CXCR7 in cardiomyocytes. (**a**) β-Arrestin recruitment assay of CXCR7 showing that CXCL12 and TC14012 induce coupling of CXCR7 with β-arrestin in a dose-dependent manner (EC_50_: 14.8 nM and 47.4 nM, respectively). Replicate samples are derived from independent HEK293 cells (n = 3). Data are shown as the mean ± SEM. (**b**) Immunoblot analysis of phosphorylated ERK (pERK) and total ERK (tERK) in HEK293 cells transfected with a CXCR7 expression plasmid at various time points after stimulation with CXCL12 (100 nM). (**c**) Quantitative data of the results shown in (**b**). n = 4. Data are shown as the mean ± SEM. Significance was calculated by ANOVA followed by the Bonferroni procedure; ***P < 0.001. Note that ERK was activated upon stimulation with CXCL12, and activity peaked at 5 min. (**d**) Immunoblot analysis of pERK/tERK in HEK293 cells transfected with various amounts of a CXCR7 expression plasmid with CXCL12 (100 nM) or vehicle (veh). Note that ERK was activated in a CXCR7 expression plasmid-dose-dependent manner under stable CXCL12 stimulation. (**e**) Quantitative data of the results shown in (**d**). n = 3. Data are shown as the mean ± SEM. Significance was calculated by one-way analysis of variance (ANOVA) followed by the Bonferroni procedure; *P < 0.05, **P < 0.01, ***P < 0.001. (**f**) *Cxcr7* mRNA expression in cardiomyocytes and fibroblasts from primary culture of neonatal rat hearts. Data are shown as the mean ± SEM. Significance was calculated by an unpaired *t*-test. **P < 0.01. (**g**) Immunoblot analysis of pERK and tERK in primary culture of NRCMs at various time points upon stimulation with the CXCR7-specific agonist, TC14012. (**h**) Quantitative data of the results shown in (**g**). n = 3. Data are shown as the mean ± SEM. Significance was calculated by ANOVA followed by the Bonferroni procedure; **P < 0.01, ***P < 0.001.

