# Supplementary material for: CXCR7 ameliorates myocardial infarction as a β-arrestin-biased receptor
Source: Sci Rep. 2021 Feb 9;11:3426. doi: 10.1038/s41598-021-83022-5 (PMC7873251; doi:10.1038/s41598-021-83022-5)
Supplement: Supplementary file 1 — Supplementary Information. [file 41598_2021_83022_MOESM1_ESM.pdf]

CXCR7 ameliorates myocardial infarction as a β-arrestin-biased receptor

Masato Ishizuka[1], Mutsuo Harada*[1, 2], Seitaro Nomura[1], Toshiyuki Ko[1], Yuichi Ikeda[1], Jiaxi Guo[1], Satoshi Bujo[1], Haruka Yanagisawa-Murakami[1], Masahiro Satoh[1], Shintaro Yamada[1], Hidetoshi Kumagai[1, 3], Yoshihiro Motozawa[1], Hironori Hara[1], Takayuki Fujiwara[1], Tatsuyuki Sato[1], Norifumi Takeda[1], Norihiko Takeda[1], Kinya Otsu[4], Hiroyuki Morita[1], Haruhiro Toko[1, 3] & Issei Komuro*[1]

[1]Department of Cardiovascular Medicine, Graduate School of Medicine, The University of Tokyo, Tokyo, Japan.

[2]Department of Advanced Clinical Science and Therapeutics, Graduate School of Medicine, The University of Tokyo, Tokyo, Japan.

[3]Department of Advanced Translational Research and Medicine in Management of Pulmonary Hypertension, Graduate School of Medicine, The University of Tokyo, Tokyo, Japan.

[4]The School of Cardiovascular Medicine and Sciences, King's College London British Heart Foundation Centre of Excellence, London, UK.

Supplementary Figure S1

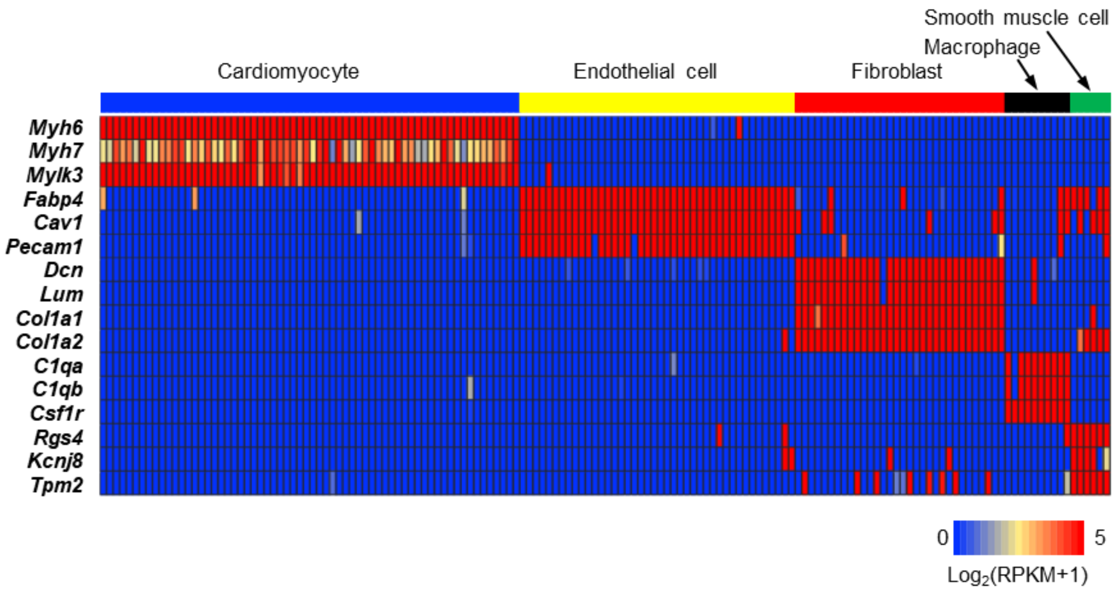

Supplementary Figure S1. Cell-type-specific marker genes confirm the precision of cell clustering by single-cell RNA-sequencing analysis. The results of RNA-sequencing cell-clustering analysis recapitulate the cell characteristics identified by cell-type specific gene markers, including *Myh6*, *Myh7*, and *Mylk3* for cardiomyocytes, *Fabp4*, *Cav1*, and *Pecam1* for endothelial cells, *Dcn*, *Lum*, *Col1a1*, and *Col1a2* for fibroblasts, *C1qa*, *C1qb*, and *Csf1r* for macrophages, and *Rgs4*, *Kcnj8*, and *Tpm2* for smooth muscle cells.

Supplementary Figure S2

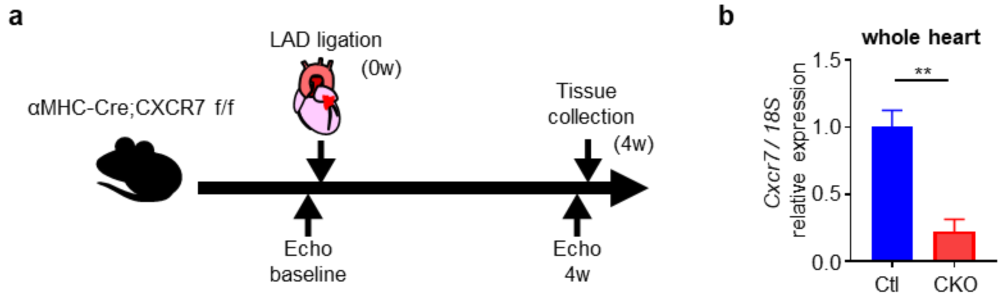

Supplementary Figure S2. Cardiomyocytes are the major cell type expressing CXCR7. (a) Design of the experiment using αMHC-Cre; CXCR7$^{f/f}$ mice. Tissues were collected four weeks after left ascending artery ligation. Transthoracic echocardiography was performed before ligation and four weeks after ligation. **(b)** *Cxcr7* gene expression in control (Ctl) and cardiomyocyte-specific *Cxcr7* knockout (CKO) mouse hearts. Ctl, n = 3; CKO, n = 5. Data are shown as the mean ± SEM. Significance was calculated by an unpaired *t*-test; ***P* < 0.001.

Supplementary Figure S3

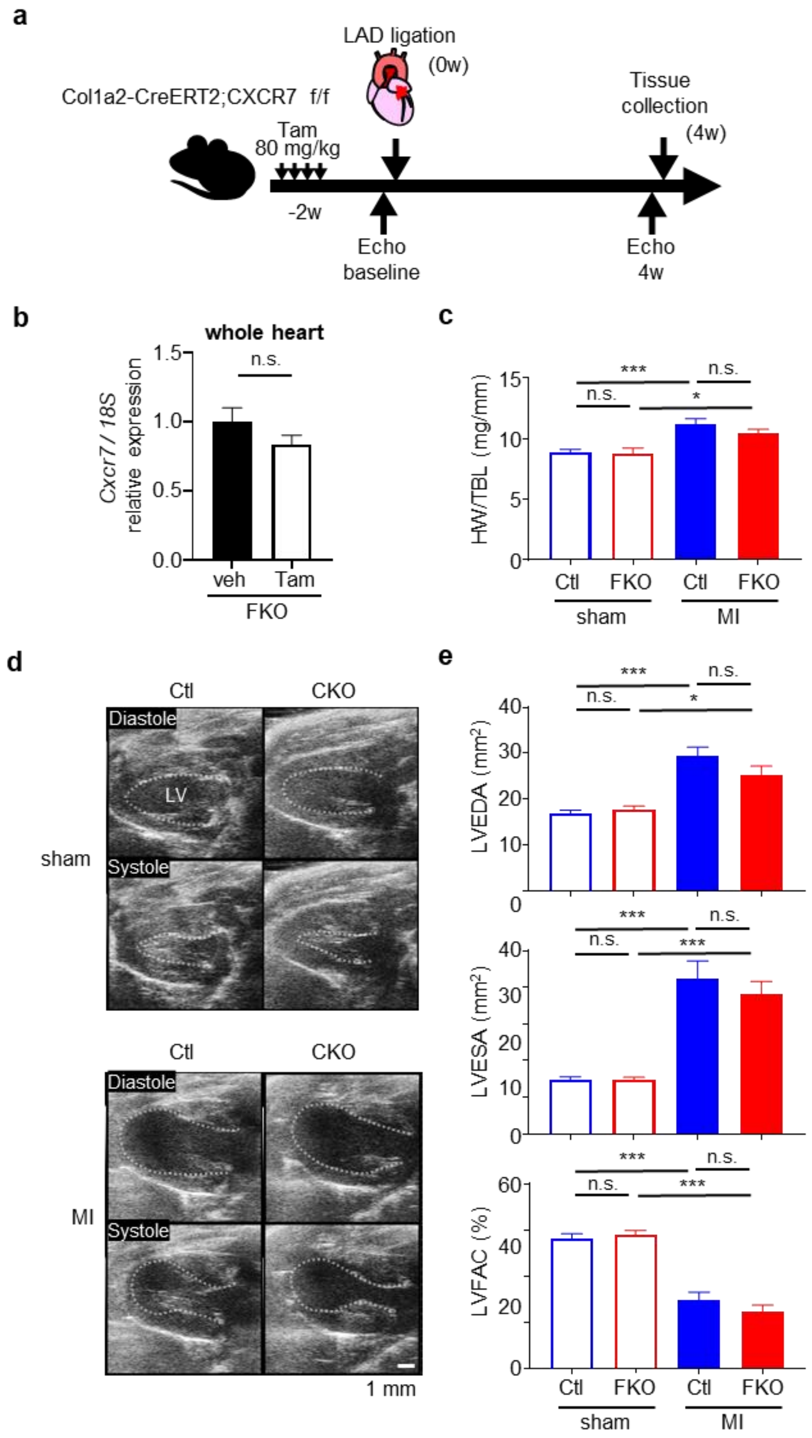

Supplementary Figure S3. Fibroblast-specific *Cxcr7*-deleted mice demonstrate no different phenotypes from control mice after myocardial infarction. (a) Design of the experiment using Col1a2-CreERT2; CXCR7$^{f/f}$ mice.

Left ascending artery ligation was operated two weeks after oral tamoxifen administration (80 mg/kg, 4 days). Tissues were collected four weeks after ligation. Transthoracic echocardiography was performed before ligation and four weeks after ligation. **(b)** *Cxcr7* gene expression with vehicle (veh) or tamoxifen (Tam) administration in fibroblast-specific *Cxcr7* knockout (FKO) mouse hearts. veh, n = 5; Tam, n = 5. Data are shown as the mean ± SEM. Significance was calculated by an unpaired *t*-test. **(c)** Heart weight-to-tibia length ratio (HW/TBL) of myocardial infarction mice. sham-Ctl, n = 7; sham-FKO, n = 5; MI-Ctl, n = 8; MI-FKO, n = 6. Data are shown as the mean ± SEM. Significance was calculated by one-way analysis of variance (ANOVA) followed by the Bonferroni procedure. ***$P < 0.001$. **(d)** Representative B-mode images of transthoracic echocardiography at diastole and systole of sham and infarcted hearts in Ctl and FKO mice. The dashed line indicates the endocardial surface of the left ventricular (LV) cavity. **(e)** Left ventricular end-diastolic area (LVEDA), end-systolic area (LVESA), and fractional area change (LVFAC) assessed by echocardiography 4 weeks after myocardial infarction. sham-Ctl, n = 7; sham-FKO, n = 5; MI-Ctl, n = 8; MI-FKO, n = 6. Data are shown as the ± SEM. Significance was calculated by one-way ANOVA followed by the Bonferroni procedure. *$P < 0.05$, **$P < 0.001$

Supplementary Figure S4

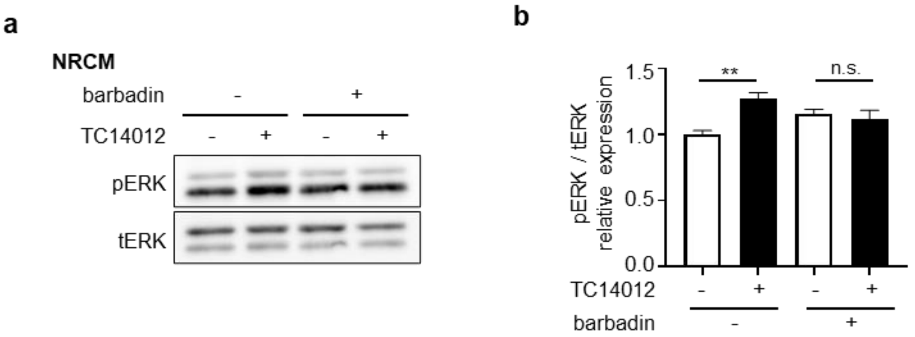

Supplementary Figure S4. Inhibition of β-arrestin signaling abolishes TC14012-induced ERK activation. (a) Immunoblot analysis of pERK and tERK in primary cultures of NRCMs after a 30-min pretreatment with dimethyl sulfoxide (vehicle) or barbadin (50 μM) and after 10 min of stimulation with phosphate buffered saline (vehicle) or TC14012 (3 μM). **(b)** Quantitative data of the results shown in (a). n = 4. Data are shown as the mean ± SEM. Significance was calculated by ANOVA followed by the Bonferroni procedure; $^{**}P < 0.001$.

Supplementary Figure S5

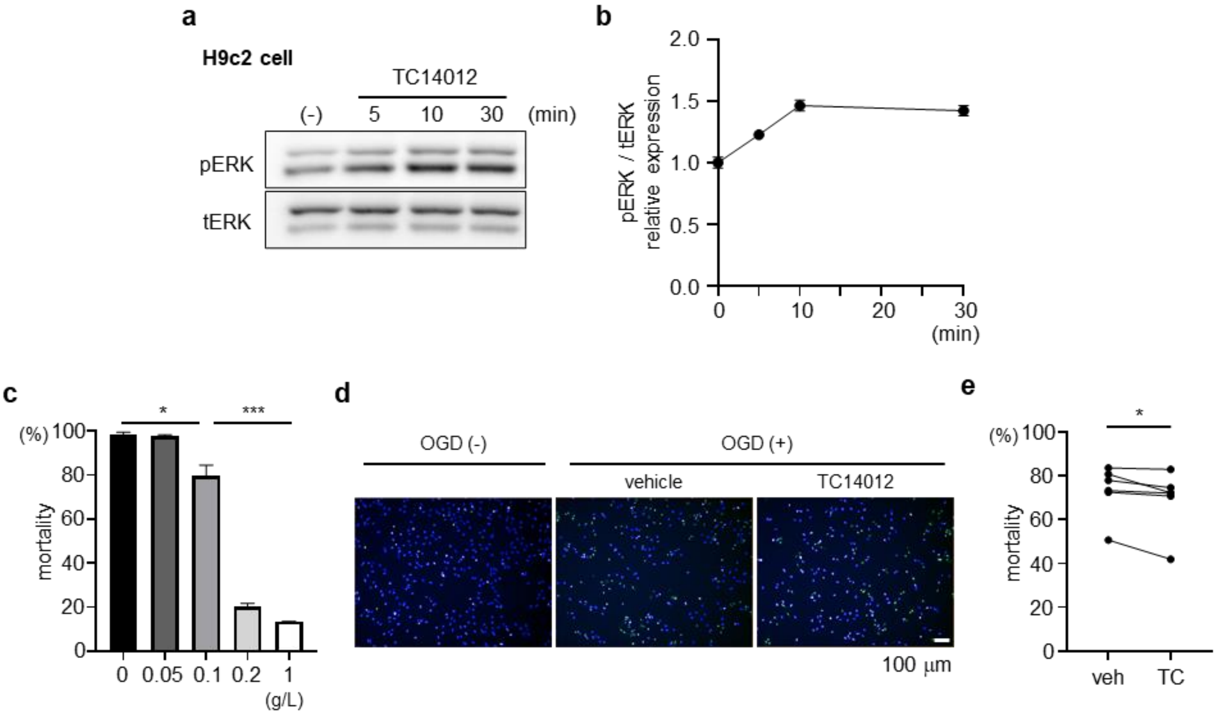

Supplementary Figure S5. TC14012 activates ERK and protects against oxygen-glucose deprivation (OGD) in H9c2 cells. (a) Immunoblot analysis of pERK and tERK in H9c2 cells at various time points upon stimulation with TC14012. **(b)** Quantitative data of the results shown in (a). n = 3. Data are shown as the mean ± SEM. Significance was calculated by ANOVA followed by the Bonferroni procedure; $**P < 0.001$ **(c)** Mortality of H9c2 cells after OGD (24 h anoxia, various glucose concentrations). **(d)** Representative images of H9c2 cells after OGD (24 h anoxia, glucose 0.1 g/L), treated with vehicle or TC14012, and stained with Hoechst (blue, all nuclei) and SYTOX (green, dead nuclei). **(e)** Mortality of H9c2 cells after OGD with vehicle or TC14012 (3 μM) as shown in (d). n = 6. Data are shown as paired data points. Significance was calculated by a paired *t*-test. $*P < 0.05$.

Supplementary Figure S6

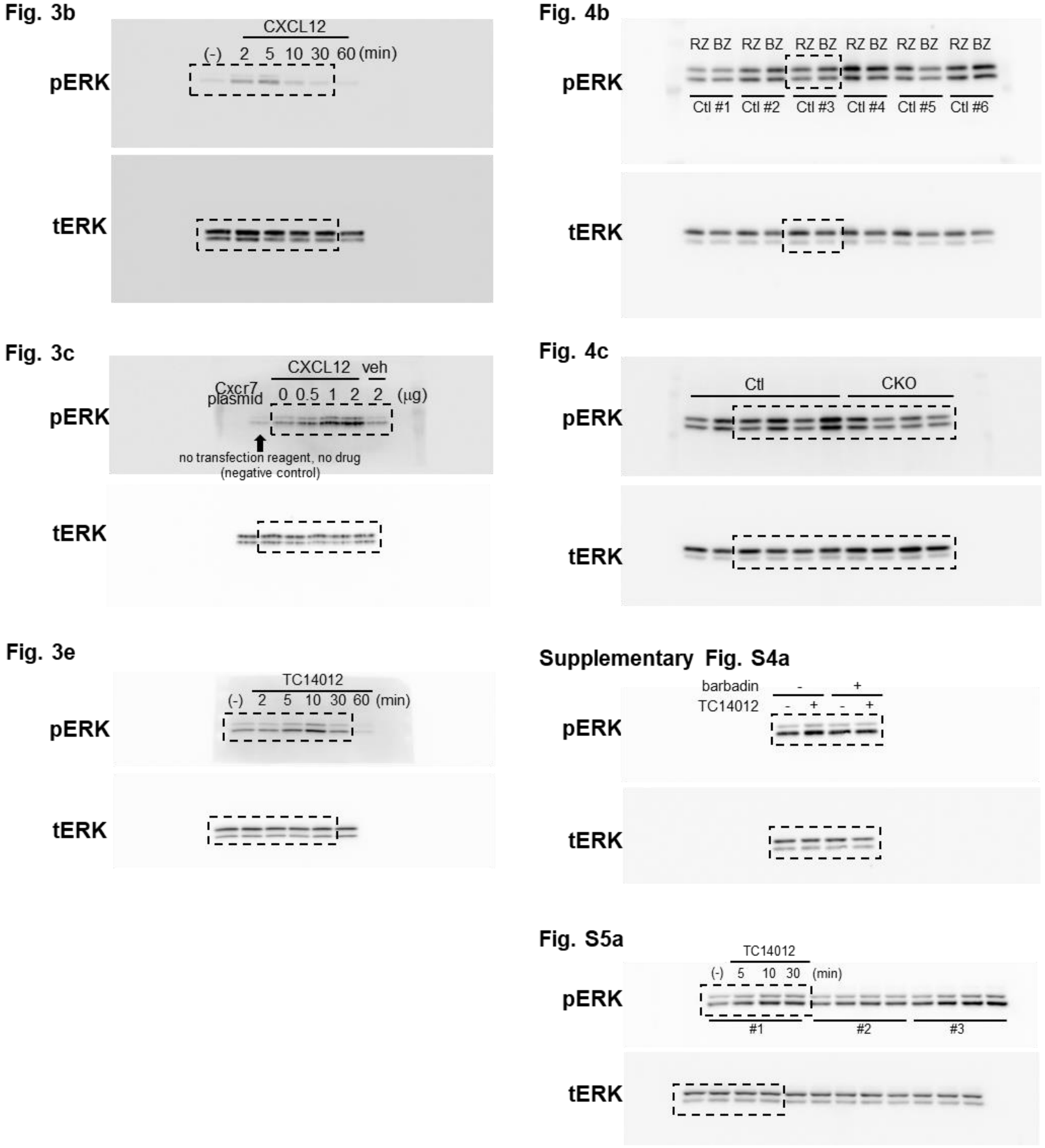

Supplementary Figure S6. Original immunoblot images for Figs. 3, 4, S4, S5. The dashed squares indicate images shown in Figs. 3, 4, S4, S5.
